# Assessing index CD4 and associated outcomes at 1-year in a tertiary HIV clinic, KwaZulu-Natal

**DOI:** 10.4102/safp.v66i1.5803

**Published:** 2024-01-25

**Authors:** Zanele R. Moya, Somasundram Pillay, Nombulelo Magula

**Affiliations:** 1 Division of Internal Medicine, School of Clinical Medicine, College of Health Sciences, University of KwaZulu-Natal, Durban, South Africa

**Keywords:** human immunodeficiency virus infection, universal test and treat, cluster of differentiation 4 count, pre-test and treat era, antiretroviral treatment initiation, opportunistic infection, tuberculosis

## Abstract

**Background:**

Human immunodeficiency virus (HIV) management guidelines have evolved from initiating therapy at CD4 counts of ≤ 200 cells/m^3^ to implementing universal test and treat (UTT). This study aimed to assess whether in clinical practice, patients are presenting with higher baseline CD4 counts, describe the incidence of opportunistic infections and the proportion that achieved viral suppression.

**Methods:**

A retrospective cohort design with convenience sampling was conducted. Cohort 1 included patients initiated on antiretroviral therapy (ART) between 01 January 2014 and 31 December 2014, when criteria were set at CD4 count ≤ 350 cells/mm^3^. Cohort 2 included patients initiated on ART between 01 January 2019 and 31 December 2019, during the UTT era.

**Results:**

At ART initiation, the median CD4 cell was 170 cells/mm^3^ (interquartile range [IQR]: 85.5–287) in Cohort 1 cells/mm^3^ and 243 cells/mm^3^ (IQR: 120–411) in Cohort 2. Tuberculosis was the predominant OI in the group with CD4 cell count ≤ 200 cells/m^3^ in both Cohort 1 (26.8%) and Cohort 2 (27.9%), *p* = 0.039. At 1 year, virological suppression was achieved in only 77.7% and 84.7% of Cohorts 1 and 2 patients.

**Conclusion:**

A notable portion of patients at King Edward VIII Hospital’s HIV clinic commenced ART with CD4 counts significantly below the recommended guideline thresholds.

**Contribution:**

The research revealed a delay in initiating ART. A comprehensive reevaluation is essential to pinpoint the factors contributing to this delay and to devise customised interventions.

## Introduction

The CD4 cell count is used as a marker of immune status and, in the past, was used as a guide for initiating antiretroviral therapy (ART) in people living with human immunodeficiency virus (PLHIV). Over the past years, HIV management guidelines have shifted from initiating therapy at criteria of a CD4 cell count ≤ 200 cells/m^3^ to universal test and treat (UTT). It was anticipated that only a small proportion of patients would present with a baseline CD4 count ≤ 200 cells/m^3^ and/or manifestations of opportunistic infections.

### Antiretroviral therapy background

After the emergence of the HIV epidemic, extensive research was conducted to explore potential pharmacological treatments. Initial clinical investigations focused on assessing the effectiveness of zidovudine as a monotherapy regimen to reduce toxicity.^1,2^ However, this approach yielded only temporary therapeutic benefits and failed to demonstrate a significant advantage in terms of patient survival.^3,4^

Subsequently, the Italy, Netherlands, Canada, Australia (INCA) study revealed that triple combination therapy was more effective in continuously reducing HIV viral load.^5^ These innovative therapeutic options marked a transformative shift in the clinical management of HIV, turning it from a subacute disease into a chronic condition.^6^

A positive immunological response to ART is associated with a significant increase in CD4 T lymphocyte count, typically within the 100 cells/mm^3^ – 250 cells/mm^3^ range. This increase, in turn, results in a reduced risk of developing opportunistic infections, particularly when CD4 levels reach higher thresholds.^7^

Antiretroviral therapy used in the treatment of HIV infections is categorised into distinct classes based on their molecular mechanisms and resistance profiles. These classes include nucleoside reverse transcriptase inhibitors (NRTIs), non-nucleoside reverse transcriptase inhibitors (NNRTIs), integrase inhibitors (INSTIs), protease inhibitors (PIs), fusion inhibitors (Fis) and co-receptor antagonists (CoRAs).^6^

The side effect profile of NRTI therapy is primarily a result of these drugs inhibiting host mitochondrial deoxyribonucleic acid (DNA) polymerase, which can lead to adverse effects such as lactic acidosis, subcutaneous lipoatrophy, peripheral neuropathy and pancreatitis. Stavudine^®^, in particular, is associated with a higher risk of these side effects.^8^

Non-nucleoside reverse transcriptase inhibitors are known to be associated with side effects such as skin reactions and hepatitis, typically occurring early in the course of therapy.

The major complications associated with PI therapy are the progressive accumulation of visceral fat and metabolic disturbances.^9,10^

One of the key challenges in HIV management guidelines has been determining the optimal timing for ART. Early initiation has been linked to several benefits, including the preservation of the immune system, a reduced risk of HIV transmission, and more rapid viral replication suppression. However, early initiation also comes with potential risks, including adverse drug effects that can affect a patient’s quality of life.^11^

### Antiretroviral therapy guidelines

The South African Department of Health (DoH) guidelines follow the World Health Organization (WHO) recommendations. The first DoH guidelines in 2004 adopted treatment initiation at a CD4 count ≤ 200 cells/mm^3^.^12,13^ Initiating therapy before the CD4 cell count falls below 200 cells/mm^3^ provided clinical benefit. Nevertheless, the precise threshold above 200 cells/mm^3^ for initiating treatment had not been definitively identified. Furthermore, the survival outcomes for patients who initiated treatment at higher CD4 cell counts had not been firmly established,^14^ and it was believed that exceeding this target could lead to potential side effects associated with drug exposure.^15^

As a result, the initial guideline recommended adhering to the specified CD4 cell count threshold. The Comprehensive Program for Research in Aids (CIPRA HT 001) randomised study showed that delaying treatment in patients with a CD4 cell count between 200 cells/mm^3^ and 350 cells/mm^3^ resulted in long-term immune dysfunction and a persistent increase in tuberculosis (TB).^16^ This led to a policy change to shift the initiation of therapy to a CD4 cell count of 350 cells/mm^3^ or below.^17^ In 2015, the WHO revised ART initiation recommendations to initiate all adults living with HIV at any CD4 cell count based on supporting evidence from multiple trials.^18^ The Trial of Early Antiretroviral and Isoniazid Prevention Therapy in Africa (TEMPRANO) showed a lower risk of death or HIV-related illness with earlier ART initiation than with deferred treatment.^19^ The INSIGHT strategic timing of antiretroviral treatment (START) study showed a significant reduction in mortality and morbidity in patients initiated on ART with a CD4 cell count greater than 500 cells/mm^3^. The benefit outweighed the risk of drug toxicity.^20^ The HIV Prevention Trial Network (HPTN 052) demonstrated that early initiation of ART reduced sexual transmission of HIV in serodiscordant couples, revealing that the largest benefit in testing and treating was in preventing transmission.^21,22^

The United States National Institutes of Health (NIH) and the WHO implemented the UTT guidelines in 2016 with the aim of early detection of HIV infection to lower HIV transmission at the community level.^23,24^

Adopting the UTT guidelines in September 2016, South Africa aimed to detect HIV infection early, thereby reducing HIV transmission at the community level. This study was prompted by the observation of hospital admissions related to complications associated with HIV in a region of high HIV burden, specifically KwaZulu-Natal (KZN), South Africa.

Against this backdrop, the study’s primary objective was to assess whether, in clinical practice, patients were presenting with higher CD4 counts following the expansion of CD4 criteria. Furthermore, the study sought to describe the prevalence of opportunistic infections and evaluate the proportion of patients who achieved viral load suppression.

## Methods

### Study design

A retrospective quantitative cohort study was conducted at the King Edward VIII Hospital HIV (KEH HIV) clinic. Patient records were the primary tool that was used to collect data.

### Study setting and population

The KEH HIV clinic is based in the eThekwini District of the province of KZN. This clinic accepts patients from surrounding healthcare centres who need ART initiation and tertiary level of healthcare. All patients who met the inclusion criteria were included in the study.

### Sampling strategy

The clinic sees approximately 2810 patients monthly, of which there are around 10 new patients per month. Sample size calculations were performed for an independent *t*-test with alpha error rates set at 0.05 and statistical powers of 0.80 and 0.90. Gpower^®^ software was utilised for this purpose and recommended an overall sample size of 260. Consequently, the study aimed to review approximately 130 patient files from each period.

This was based on a convenience sampling technique whereby clinical records were reviewed from 01 January 2014 to 31 December 2014 (Period 1) and 01 January 2019 to 31 December 2019 (Period 2), meeting the inclusion criteria explained next.

### Inclusion criteria

The inclusion criteria encompassed all adult patients aged 18 and above who initiated ART at the KEH HIV clinic during two distinct periods:

- Period 1 (01 January 2014 to 31 December 2014): This period corresponded to a phase when South African national HIV guidelines recommended initiating ART when a patient’s CD4 cell count was at or below 350 cells/mm³.
- Period 2 (01 January 2019 to 31 December 2019): This timeframe aligned with the implementation of South African national HIV guidelines calling for initiating ART as soon as an HIV test yielded a positive result, regardless of CD4 count, known as the UTT era.

### Exclusion criteria

The exclusion criteria encompassed:

- Patients who had previously or were currently undergoing ART
- Patients younger than 18 years of age

### Measuring clinical outcomes

Clinical outcomes were assessed through two primary indicators: viral load suppression and CD4 cell count at the 12-month mark. Viral load suppression was defined as having fewer than 400 copies per millilitre viral copies, and this measurement was conducted at 16 weeks post-initiation as per established guidelines.^25^

### Statistical analysis

The data collected were analysed with SPSS version 28.0 (IBM Corp, Armonk, New York, United States [US]) and Stata version 16.0 (StataCorp, College Station, Texas, US). Categorical data were presented as frequencies and percentages and compared utilising chi-square tests (goodness-of-fit for single variables and test-of-independence for bivariate data). Descriptive statistics (mean and standard deviation) were used to describe the continuous collected data. Continuous variable group means were compared using the Kruskal–Wallis test. A *p*-value of < 0.05 was regarded as statistically significant. All *p*-values are chi-square values unless otherwise specified.^26^

### Ethical considerations

The Biomedical Research Ethics Committee of the University of KwaZulu-Natal granted ethical approval for this study (BREC/00001701/2020). Approval was obtained from the hospital and KZN provincial Department of Health.

## Results

The total study sample comprised 241 patients (112 vs. 129 in Cohorts 1 and 2, respectively). Most patients were between 26 and 35 (36.6% vs. 51.9%, *p* < 0.001 in Cohorts 1 and 2, respectively). The patients in the study were predominantly female, with the racial distribution being primarily black African (see Table 1).

**TABLE 1:** Socio-demographic details of the study sample (*n* = 241).

Demographic variable	Cohort 1 (*n* = 112)		*p*	Cohort 2 (*n* = 129)		*p*	*p*-value for both cohorts
	Count	%		Count	%		
**Age (years)**	-	-	< 0.001	-	-	< 0.001	< 0.001
18–25	4	3.6	-	19	14.7	-	-
26–35	41	36.6	-	67	51.9	-	-
36–45	34	30.4	-	23	17.8	-	-
46–55	23	20.5	-	12	9.3	-	-
> 55	10	8.9	-	8	6.2	-	-
**Sex**	-	-	0.059	-	-	0.001	0.428
Male	46	41.1	-	46	35.9	-	-
Female	66	58.9	-	82	64.1	-	-
**Race**	-	-	< 0.001	-	-	< 0.001	0.463
Black people	110	98.2	-	122	94.6	-	-
Indian people	1	0.9	-	4	3.1	-	-
Mixed race people	1	0.9	-	1	0.8	-	-
White people	0	0.0	-	2	1.6	-	-

Table 2 presents the differences in median CD4 cell counts between two cohorts at two time points: baseline and after 1 year. At baseline, Cohort 1 had a lower median CD4 cell count of 170 cells/mm^3^ (interquartile range [IQR]: 85.5–287) than Cohort 2, with a median count of 243 cells/mm^3^ (IQR: 120–411). After 1 year, the CD4 cell counts increased to 332.50 cells/mm^3^ (IQR: 228–517.5) and 372 cells/mm^3^ (IQR: 222–579) in Cohorts 1 and 2, respectively. However, the difference between the two cohorts was not statistically significant (*p* = 0.680).

**TABLE 2:** Each cohort’s median CD4 count at baseline and 1 year.

Variable	Cohort 1 (*n* = 112)		*p*	Cohort 2 (*n* = 129)		*p*	*p*-value for both cohorts
	Median	IQR		Median	IQR		
CD4 (cells/mm^3^) at baseline	170	85.5–287. 0	< 0.001	243.0	120–411	< 0.001	0.001
CD4 (cells/mm^3^) at 1 year	332.50	228.0–517		372.0	222.0–579.0	-	0.680

IQR, interquartile range.

Table 3 presents the frequency distribution of CD4 cell counts at baseline, the 1-year follow-up for Cohorts 1 and 2, and the corresponding *p*-values for both cohorts. In Cohort 1, at baseline, 57.1% of patients had a CD4 cell count of less than 200 cells/mm^3^ (*p* < 0.001). After 1 year of treatment, there was a notable improvement, with 45.5% of patients achieving CD4 cell counts above 350 cells/mm^3^. The differences observed between the CD4 categories in Cohort 1 were statistically significant (*p* < 0.001). In Cohort 2, at baseline, 41.9% of patients had a CD4 cell count of fewer than 200 cells/mm^3^, which was higher than the proportion of patients with CD4 cell counts between 200 cells/mm^3^ and 350 cells/mm^3^ (23.3%), and those with CD4 cell counts above 350 cells/mm^3^ (34.9%) (*p* = 0.033). After 1 year of treatment, most patients achieved CD4 cell counts above 350 cells/mm^3^ (55.8%).

**TABLE 3:** Frequency table describing CD4 counts at baseline and 1 year.

CD4 category (cells/mm^3^)	CD4 at index presentation				*p*-value for both cohorts	CD4 at 1 year				*p*-value for both cohorts
	Cohort 1 (*n* = 112)		Cohort 2 (*n* = 129)			Cohort 1 (*n* = 112)		Cohort 2 (*n* = 129)		
	Count (%)	*p*	Count (%)	*p*	Count (%)	*p*	Count (%)	*p*
< 200	64	< 0.001	54	0.033	< 0.001	24	0.008	32	< 0.001	0.055
	57.1%	-	41.9%	-	-	21.4%	-	24.8%	-	-
200–350	41	-	30	-	-	37	-	25	-	-
	36.6%	-	23.3%	-	-	33.0%	-	19.4%	-	-
> 350	7	-	45	-	-	51	-	72	-	-
	6.3%	-	34.9%	-	-	45.5%	-	55.8%	-	-

The study captured opportunistic infection present at diagnosis and during the period under review. It showed TB (both pulmonary and disseminated) as the predominant OI with the highest prevalence of TB infections in the group with CD4 cell count < 200 mm^3^ in both Cohort 1 (29 cases) and Cohort 2 (36 cases), *p* = 0.039 (see Table 4).

**TABLE 4:** The frequency of opportunistic infections and HIV-associated diseases in each cohort.

Variables	Cohort 1 (CD4 cells/mm^3^)			Cohort 2 (CD4 cells/mm^3^)			*p*
	< 200	200–350	> 350	< 200	200–350	> 350	
Tuberculosis	23	7	0	26	4	6	0.039
Recurrent Severe bacterial pneumonia	1	0	0	2	0	0	-
Candidiasis	2	0	1	1	0	0	-
HIV-associated malignancy†	1	1	1	1	0	0	0.513
Pneumocystis jiroveci pneumonia	1	0	0	0	0	0	-
Herpes zoster	1	1	0	1	1	2	0.472
Other‡	6	1	1	5	0	2	0.506
None	29	32	4	21	25	35	< 0.001

† , Cervical cancer, Kaposi sarcoma, non-Hodgkin’s lymphoma.

‡ , Syphilis, cryptococcal meningitis.

Table 5 demonstrates that in Cohort 1, 77.7% and Cohort 2, 81.4% of the patients achieved virological suppression at 1 year post-ART initiation. This was statistically significant within each cohort (*p* < 0.001); however, there was no significant difference between the two cohorts (*p* = 0.475).

**TABLE 5:** Outcomes in viral load suppression at 1 year within each cohort.

Viral load at 12 months	Cohort 1 (*n* = 112)		*p*	Cohort 2 (*n* = 129)		*p*	Accumulative total (*n* = 241)		*p*
	*n*	%		*n*	%		*n*	%	
Suppressed	87	77.7%	*p* < 0.001	105	81.4%	*p* < 0.001	192	79.7%	*p* = 0.475
Non suppressed	25	22.3%	-	24	18.6%	-	49	20.3%	-

Suppressed viral load: < 400 copies/mL at 6 months.

Not suppressed viral load: > 400 copies/mL at 6 months.

## Discussion

This study presented an examination of individuals who commenced ART for HIV infection at distinct target CD4 levels, as suggested by the prevailing HIV treatment guidelines during that period.

Importantly, this sample of patients’ index testing and diagnosis for HIV occurred at a tertiary institution after referral from surrounding healthcare centres to various specialities. The study scrutinised these patients’ initial CD4 cell counts upon their presentation and subsequently assessed their CD4 levels again after 1 year. Additionally, the study assessed the prevalence of virological suppression among these patients at 1 year. Furthermore, the investigation characterised the profile of opportunistic infections observed in this sample of HIV-positive individuals.

South Africa continues to grapple with a substantial HIV epidemic, with KZN emerging as one of the provinces facing a disproportionate burden of the disease, particularly among the younger population.^27^

Our study revealed that the majority of patients in both Cohorts 1 and 2 who initiated therapy fell within the age range of 26–35 years, constituting 36.6% and 51.9% of the respective cohorts. This observed prevalence aligned with the findings of the household-based HIV serosurvey, which documented a higher incidence of infections among individuals aged 25–34 years, with the highest infection rates observed among 26-year-old females.^28^ The gender distribution in both cohorts primarily comprised females, reflecting the gender distribution patterns observed in South Africa.^29^

Extensive research indicates that factors such as increased female participation in rural-to-urban migration and socio-economic challenges such as unemployment may contribute to elevated infection rates among women.^29^ Regarding racial demographics, the study population predominantly consisted of black individuals, accounting for 98.2% in Cohort 1 and 94.6% in Cohort 2, aligning with national epidemiological data and the specific geographical context of the study.^30^

Over more than two decades, the measurement of CD4 cell count has emerged as a pivotal tool in comprehending the progression of HIV disease and assessing the susceptibility to opportunistic infections.^31^ The development of guidelines concerning initiating ART at higher CD4 cell counts has been influenced by evidence suggesting that deferring treatment until CD4 cell counts decline below 200 cells/mm^3^ is linked to increased mortality rates.^32^ These guidelines are formulated with consideration for a population that undergoes annual HIV testing to commence treatment before the onset of immunodeficiency.^33^

In Cohort 1, the median CD4 cell count at the initiation of ART was measured at 170.0 mm^3^ (IQR: 85.50 mm^3^ – 287.0 mm^3^, while in Cohort 2, it was recorded as 243.0 mm^3^ (IQR: 120.00 mm^3^ – 411.0 mm^3^).

Despite the implementation of higher CD4 cell count targets and the era of UTT, the study revealed that the majority of patients in both cohorts (57.1% in Cohort 1 and 41.9% in Cohort 2) had CD4 cell counts below 200 cells/mm^3^ at the time of treatment initiation. A meta-analysis conducted in sub-Saharan Africa, focusing on CD4 cell counts at treatment initiation, indicated that there has been no discernible change in the trend of CD4 cell counts over the past decade, irrespective of the specified threshold.^34^ This persistent lack of change may be attributable to factors such as reduced uptake of HIV testing because of prevailing HIV-related stigma, as well as geographic- and transportation-related barriers.^35,36^

Cohort 2 exhibited a substantially greater proportion of patients with CD4 cell counts exceeding 350 mm^3^ (34.9%) than Cohort 1 (6.3%). This discrepancy was anticipated, as Cohort 2 represented the UTT era, wherein treatment initiation was not solely based on CD4 cell count thresholds. In contrast, patients in Cohort 1 were initiated on treatment because of alternative indications, such as co-infection with TB or other factors.

The OIs pose a significant threat to the health and survival of individuals living with HIV. Our study revealed a higher prevalence of OIs among patients with CD4 cell counts below 200 cells/mm^3^, with 31.25% in Cohort 1 and 25.58% in Cohort 2 being affected. Among the observed OIs, TB infection was the most commonly occurring, with higher numbers observed in the more immunodeficient group (30 cases in Cohort 1 vs. 36 cases in Cohort 2). This finding aligned with previous retrospective studies identifying TB and oral candidiasis as the two most common OIs.^37,38^ A prospective cohort study conducted by Murphy et al. at Mc Cord Hospital in KZN also reported TB as the predominant OI, accounting for 76% of cases (43). Our study additionally identified other OIs, including herpes zoster (2.9%), bacterial pneumonia (1.2%), candidiasis (2.1%), pneumocystis jiroveci pneumonia (0.4%), and HIV-associated malignancies such as non-Hodgkin’s lymphoma, cervical cancer, and Kaposi’s sarcoma (1.7%). The study also documented cases of syphilis and cryptococcal meningitis (7.1%) among the patients. These findings underscored the diverse range of OIs that can occur in HIV-positive individuals with compromised immune function and emphasised the importance of screening for such infections.

In our study, we found that at the 1-year mark, the median CD4 cell count was 332.50 cells/mm^3^ (IQR: 228.00–517.50) in Cohort 1 and 372.00 cells/mm^3^ (IQR: 222.00–579.00) in Cohort 2. These results align with a study conducted by Mocroft et al., which reported a median CD4 count of 204 cells/mm^3^ (IQR: 85–330) at initiating ART. Mocroft et al. also observed an average increase of 100 cells/mm^3^ in CD4 count after 1 year of initiating ART.^39^ Notably, they found that patients with lower baseline CD4 counts exhibited a greater increase in CD4 count compared with those initiating treatment at CD4 counts above 500 cells/mm^3^.^40^ This highlighted the significance of initiating treatment at higher CD4 counts to preserve immunological function in patients.

In Cohort 1, 87 out of 112 patients (77.67%) achieved virological suppression, while in Cohort 2, 105 out of 124 patients (84.67%) achieved virological suppression. One of the United Nations Joint Programme on HIV/AIDS (UNAIDS) 90-90-90 targets aimed to achieve 90% viral suppression in individuals receiving ART by 2020, with the overall goal of curbing the HIV epidemic by 2030. Our study found no significant difference in the rates of virological suppression between the cohorts (*p* = 0.475), irrespective of their initial CD4 cell counts. However, within each cohort, there was a significant variation in the number of patients who achieved suppressed viral loads (*p* < 0.001). It is important to acknowledge that during the study period, the rates of viral suppression did not meet the target. Nevertheless, a notable proportion of patients achieved virological suppression. Factors contributing to virological failure included poor adherence to treatment and the development of opportunistic infections within our cohorts.

### Limitations of the study

This study was limited by its design, which was retrospective. The sample size was small and looked at a duration of 12 months. The two cohorts included patients from a tertiary hospital, which may not have reflected local and rural areas of KZN. A more extensive prospective study would confirm and help understand the underlying factors responsible for starting treatment late.

## Conclusion

This study revealed that, despite a change in the guidelines with the removal of CD4 counts cut-off levels of 350 cells/mm^3^ before initiating ART, a significant number of patients attending the HIV clinic in King Edward VIII Hospital still present with low CD4 counts at diagnosis, in the UTT era. These findings suggested delays in diagnosis and treatment initiation. However, the reasons for the delay were unknown and beyond the study’s scope, and further studies are required to investigate these barriers. The TB was the most typical OI infection in both cohorts, which in part may be explained by a comparable proportion of those with low CD4 cell counts being at baseline. Furthermore, viral load suppression at 1 year was below the UNAIDS target of 90% in the sample, highlighting the need to reinforce treatment adherence and investigate and address modifiable contributing factors.

## References

[1] Vella S, Schwartländer B, Sow SP, Eholie SP, Murphy RL. The history of antiretroviral therapy and its implementation in resource-limited areas of the world. AIDS. 2012;26(10):1231–1241. 10.1097/QAD.0b013e32835521a3 22706009

[2] Sandström EG, Kaplan JC. Antiviral therapy in AIDS. Drugs. 1987;34(3):372–390. 10.2165/00003495-198734030-00004 2824170

[3] Skowron G, Bozzette SA, Lim L, et al. Alternating and intermittent regimens of zidovudine and dideoxycytidine in patients with AIDS or AIDS-related complex. Ann Intern Med. 1993;118(5):321–330. 10.7326/0003-4819-118-5-199303010-00001 8094279

[4] Fischl MA, Richman DD, Hansen N, et al. The safety and efficacy of zidovudine (AZT) in the treatment of subjects with mildly symptomatic human immunodeficiency virus type 1 (HIV) infection: A double-blind, placebo-controlled trial. Ann Intern Med. 1990;112(10):727–737. 10.7326/0003-4819-112-10-727 1970466

[5] Montaner JS, Reiss P, Cooper D, et al. A randomised, double-blind trial comparing combinations of nevirapine, didanosine, and zidovudine for HIV-infected patients: The INCAS Trial. JAMA. 1998;279(12):930–937. 10.1001/jama.279.12.930 9544767

[6] Arts EJ, Hazuda DJ. HIV-1 antiretroviral drug therapy. Cold Spring Harb Perspect Med. 2012;2(4):a007161. 10.1101/cshperspect.a007161 22474613 PMC3312400

[7] Kaplan JE, Hanson D, Dworkin MS, et al. Epidemiology of human immunodeficiency virus-associated opportunistic infections in the United States in the era of highly active antiretroviral therapy. Clin Infect Disases. 2000;30(Supplement_1):S5–S14. 10.1086/313843 10770911

[8] Foster C, Lyall H. HIV and mitochondrial toxicity in children. J Antimicrob Chemother. 2008;61(1):8–12. 10.1093/jac/dkm411 17999978

[9] Magula N, Dedicoat M. Low dose versus high dose stavudine for treating people with HIV infection. Cochrane Database Syst Rev. 2015;1:CD007497. 10.1002/14651858.CD007497.pub2 25627012 PMC10862382

[10] Murphy RL. Defining the toxicity profile of nevirapine and other antiretroviral drugs. J Acquir Immune Defic Syndr. 2003;34:S15–S20. 10.1097/00126334-200309011-00004 14562854

[11] Mocroft A, Lundgren JD. Starting highly active antiretroviral therapy: Why, when and response to HAART. J Antimicrob Chemother. 2004;54(1):10–30. 10.1093/jac/dkh290 15163656

[12] World Health Organization. Scaling up antiretroviral therapy in resource-limited settings: Guidelines for a public health approach: Executive summary. Report No. 9241545674. Geneva: World Health Organization; 2002. 12154788

[13] Moorhouse M, Conradie F, Venter F. What is the role of CD4 count in a large public health antiretroviral programme? South Afr J HIV Med. 2016;17(1):1–3. 10.4102/sajhivmed.v17i1.446 PMC5843041 29568607

[14] Ying R, Granich RM, Gupta S, Williams BG. CD4 cell count: Declining value for antiretroviral therapy eligibility. Clin Infect Dis. 2016;62(8):1022–1028. 10.1093/cid/civ1224 26826372 PMC5006297

[15] Eholié SP, Badje A, Kouame GM, et al. Antiretroviral treatment regardless of CD4 count: The universal answer to a contextual question. AIDS Res Ther. 2016;13(1): 1–9. 10.1186/s12981-016-0111-1 27462361 PMC4960900

[16] Collins S, Juste J, Koenig S, et al. CD4 deficit and TB risk persist with delayed antiretroviral therapy: 5-year data from CIPRA HT-001. Int J Tuberc Lung Dis. 2015;19(1):50–57. 10.5588/ijtld.14.0217 25519790

[17] Venter FW. What is the optimal CD4 count to start antiretrovirals in HIV-infected adults? Johannesburg: Taylor & Francis; 2009.

[18] World Health Organization. Guideline on when to start antiretroviral therapy and on pre-exposure prophylaxis for HIV. Geneva: World Health Organization; 2015. 26598776

[19] Group TAS. A trial of early antiretrovirals and isoniazid preventive therapy in Africa. N Engl J Med. 2015;373(9):808–822. 10.1056/NEJMoa1507198 26193126

[20] Geffen N, Aagaard P, Corbelli GM, et al. Community perspective on the INSIGHT S trategic T iming of AntiRetroviral T reatment (START) trial. HIV Med. 2015;16(S1):10–30. 10.1111/hiv.12228 25711318 PMC4341942

[21] Cohen MS, McCauley M, Gamble TR. HIV treatment as prevention and HPTN 052. Curr Opin HIV AIDS. 2012;7(2):99. 10.1097/COH.0b013e32834f5cf2 22227585 PMC3486734

[22] Dodd PJ, Garnett GP, Hallett TB. Examining the promise of HIV elimination by ‘test and treat’in hyper-endemic settings. AIDS. 2010;24(5):729. 10.1097/QAD.0b013e32833433fe 20154580 PMC2852517

[23] Walensky RP, David Paltiel A, Losina E, et al. Test and treat DC: Forecasting the impact of a comprehensive HIV strategy in Washington DC. Clin Infect Dis. 2010;51(4):392–400. 10.1086/655130 20617921 PMC2906630

[24] Günthard HF, Saag MS, Benson CA, et al. Antiretroviral drugs for treatment and prevention of HIV infection in adults: 2016 recommendations of the International Antiviral Society–USA panel. JAMA. 2016;316(2):191–210. 10.1001/jama.2016.8900 27404187 PMC5012643

[25] Lok JJ, Bosch RJ, Benson CA, et al. Long-term increase in CD4+ T-cell counts during combination antiretroviral therapy for HIV-1 infection. AIDS. 2010;24(12):1867. 10.1097/QAD.0b013e32833adbcf 20467286 PMC3018341

[26] Cunningham JB, McCrum-Gardner E. Power, effect and sample size using GPower: Practical issues for researchers and members of research ethics committees. Evid Based Midwifery. 2007;5(4):132–137.

[27] Karim QA, Karim SSA. The evolving HIV epidemic in South Africa. Int J Epidemiol. 2002;31(1):37–40. 10.1093/ije/31.1.37 11914290

[28] Welz T, Hosegood V, Jaffar S, Bätzing-Feigenbaum J, Herbst K, Newell M-L. Continued very high prevalence of HIV infection in rural KwaZulu-Natal, South Africa: A population-based longitudinal study. AIDS. 2007;21(11):1467–1472. 10.1097/QAD.0b013e3280ef6af2 17589193

[29] Camlin CS, Hosegood V, Newell M-L, McGrath N, Bärnighausen T, Snow RC. Gender, migration and HIV in rural KwaZulu-Natal, South Africa. PLoS One. 2010;5(7):e11539. 10.1371/journal.pone.0011539 20634965 PMC2902532

[30] Chimere-Dan O. Apartheid and demography in South Africa. Afr Popul Stud. 1992;1992(7):28–38. 10.1002/hpm.4740070405 12321499

[31] Ford N, Meintjes G, Pozniak A, et al. The future role of CD4 cell count for monitoring antiretroviral therapy. Lancet Infect Dis. 2015;15(2):241–247. 10.1016/S1473-3099(14)70896-5 25467647

[32] Moore D, Hogg R, Yip B, Craib K, Wood E, Montaner J. CD4 percentage is an independent predictor of survival in patients starting antiretroviral therapy with absolute CD4 cell counts between 200 and 350 cells/μL. HIV Med. 2006;7(6): 383–3888. 10.1111/j.1468-1293.2006.00397.x 16903983

[33] Centers for Disease Control and Prevention. HIV prevention in the United States : New opportunities, new expectations [homepage on the Internet]. 2015 [cited n.d.]. Available from: https://stacks.cdc.gov/view/cdc/39420

[34] Siedner MJ, Ng CK, Bassett IV, Katz IT, Bangsberg DR, Tsai AC. Trends in CD4 count at presentation to care and treatment initiation in sub-Saharan Africa, 2002–2013: A meta-analysis. Clin Infect Dis. 2015;60(7):1120–1127. 10.1093/cid/ciu1137 25516189 PMC4366582

[35] Castro A, Farmer P. Understanding and addressing AIDS-related stigma: From anthropological theory to clinical practice in Haiti. Am J Public Health. 2005;95(1):53–59. 10.2105/AJPH.2003.028563 15623859 PMC1449851

[36] Govindasamy D, Ford N, Kranzer K. Risk factors, barriers and facilitators for linkage to antiretroviral therapy care: A systematic review. AIDS. 2012;26(16):2059–2067. 10.1097/QAD.0b013e3283578b9b 22781227

[37] Chakraborty N, Mukherjee A, Santra S, et al. Current trends of opportunistic infections among HIV-seropositive patients from Eastern India. Jpn J Infect Dis. 2008;61(1):49. 10.7883/yoken.JJID.2008.49 18219134

[38] Damtie D, Yismaw G, Woldeyohannes D, Anagaw B. Common opportunistic infections and their CD4 cell correlates among HIV-infected patients attending at antiretroviral therapy clinic of Gondar University Hospital, Northwest Ethiopia. BMC Res Notes. 2013;6:1–7. 10.1186/1756-0500-6-534 24330921 PMC3866565

[39] Murphy RA, Sunpath H, Taha B, et al. Low uptake of antiretroviral therapy and high mortality after tuberculosisTB or opportunistic infection in KwaZulu-Natal, South Africa. Int J Tuberc Lung Dis. 2010;14(7):903–908. 20550776 PMC3207641

[40] Mocroft A, Phillips AN, Gatell J, et al. Normalisation of CD4 counts in patients with HIV-1 infection and maximum virological suppression who are taking combination antiretroviral therapy: An observational cohort study. Lancet. 2007;370(9585): 407–413. 10.1016/S0140-6736(07)60948-9 17659333

